# Parathyroid Hormone versus Bisphosphonate Treatment on Bone Mineral Density in Osteoporosis Therapy: A Meta-Analysis of Randomized Controlled Trials

**DOI:** 10.1371/journal.pone.0026267

**Published:** 2011-10-12

**Authors:** Longxiang Shen, Xuetao Xie, Yan Su, Congfeng Luo, Changqing Zhang, Bingfang Zeng

**Affiliations:** Department of Orthopedic Surgery, Shanghai Sixth People's Hospital, Shanghai Jiaotong University, Shanghai, People's Republic of China; Hospital Universitario 12 de Octubre, Spain

## Abstract

**Background:**

Bisphosphonates and parathyroid hormone (PTH) represent the antiresorptive and anabolic classes of drugs for osteoporosis treatment. Bone mineral density (BMD) is an essential parameter for the evaluation of anti-osteoporotic drugs. The aim of this study was to evaluate the effects of PTH versus bisphosphonates on BMD for the treatment of osteoporosis.

**Methods/Principal Findings:**

We performed a literature search to identify studies that investigated the effects of PTH versus bisphosphonates treatment on BMD. A total of 7 articles were included in this study, representing data on 944 subjects. The pooled data showed that the percent change of increased BMD in the spine is higher with PTH compared to bisphosphonates (WMD = 5.90, 95% CI: 3.69–8.10, *p*<0.01,). In the hip, high dose (40 µg) PTH (1–34) showed significantly higher increments of BMD compared to alendronate (femoral neck: WMD = 5.67, 95% CI: 3.47–7.87, *p*<0.01; total hip: WMD = 2.40, 95%CI: 0.49–4.31, *p*<0.05). PTH treatment has yielded significantly higher increments than bisphosphonates with a duration of over 12 months (femoral neck: WMD = 5.67, 95% CI: 3.47–7.86, *p*<0.01; total hip: WMD = 2.40, 95% CI: 0.49–4.31, *P*<0.05) and significantly lower increments at 12 months (femoral neck: WMD = −1.05, 95% CI: −2.26–0.16, *p*<0.01; total hip: WMD: −1.69, 95% CI: −3.05–0.34, *p*<0.05). In the distal radius, a reduction in BMD was significant between PTH and alendronate treatment. (WMD = −3.68, 95% CI: −5.57–1.79, *p*<0.01).

**Discussion:**

Our results demonstrated that PTH significantly increased lumbar spine BMD as compared to treatment with bisphosphonates and PTH treatment induced duration- and dose-dependent increases in hip BMD as compared to bisphosphonates treatment. This study has also disclosed that for the distal radius, BMD was significantly lower from PTH treatment than alendronate treatment.

## Introduction

Osteoporosis is a common skeletal disease characterized by low bone mass and deterioration in bone micro-architecture, which induces bone fragility and increased risk of fracture [1]. Increasing bone mass and improving bone architecture and strength reduces skeletal fragility and the risk of fracture and are the optimal treatments for osteoporosis.

Antiresorptive agents, such as bisphosphonates, are the most widely used group of drugs for osteoporosis treatment [2], [3], [4]. Bisphosphonates directly reduce the number of active osteoclasts by inhibiting their recruitment and also by inhibiting the osteoclast-stimulating activity of osteoblasts [5], [6]. bisphosphonate therapy normalizes bone turnover, reduces the number of bone remodeling units, restores the balance of bone remodeling, prevents bone loss and deterioration of bone structure and reduces fracture risk in patients with osteoporosis [6], [7], [8].

Parathyroid hormone (PTH) is used clinically as an anabolic agent [9], [10], [11], [12], [13]. Two forms of recombinant human PTH have been evaluated: teriparatide, the 34 residue amino-terminal fragment of human PTH (1–34) and the intact 84-amino acid form of PTH (1–84), which is marketed as Preotact® [14]. PTH directly increases osteoblast production rate and inhibits apoptosis of osteoblasts, thereby leading to a rapid increase in skeletal mass as well as improvement of bone micro-architecture and strength [15].

A decrease in bone mineral density (BMD) is a significant risk factor for fracture and is of similar importance in both women and men [16], [17]. The measurement of BMD is a major determinant of fracture and an essential parameter for the evaluation of anti-osteoporotic drugs used in clinical therapy. As more studies comparing the effects of PTH and bisphosphonates on BMD in patients with osteoporosis are now becoming available, we decided to perform a meta-analysis on the effects of PTH and bisphosphonates on BMD for the treatment of osteoporosis. Our main goal was to study the effects of PTH and bisphosphonates on BMD separately at various skeletal sites (lumbar spine, total hip, femoral neck and distal radius).

## Methods

### Search Strategy

This meta-analysis followed the PRISMA statement guidelines [18]. A literature search was performed on August 17, 2010 and an updated search was performed on 14 April 2011 using the phrase, “parathyroid hormone AND bisphosphonate AND osteoporosis” with the limits “humans” and “randomized controlled trial”. A second search was performed using the phrase, “parathyroid hormone AND bisphosphonate AND bone mineral” with the limits “humans” and “randomized controlled trial” using PubMed (1990–2010), Ovid's MEDLINE (1990–2010), MEDLINE In Process &. Other Non-Indexed Citations (1990-2010), Web of Knowledge and EMBASE (1991–2010). Further searches using the same keywords and limitations did not provide additional references. We also performed a search of the Cochrane Central Register of Controlled Trials and a conference abstract search of the Journal of Bone and Mineral Research. Review articles were also scanned to find additional eligible studies. In addition, reference lists of all original articles and previous systematic reviews were hand searched for other relevant papers. The searches were not restricted to English language literature. Duplicates were removed. Information was carefully extracted from all eligible publications independently by two of the authors of the present study (LS and XX). Differences in the extraction of data were inspected by a third investigator (CL). All the searched studies were retrieved and their references were checked for other relevant publications. The search results were then screened on the basis of the following inclusion criteria: (a) randomized controlled studies with a duration of at least 6 months, (b) The active treatment arm of the study had to include PTH and bisphosphonate, and (c) Studies on patients with postmenopausal or gonadal osteoporosis. Exclusion criteria included non-randomized trials or duration of less than 6 months and studies on any secondary osteoporosis (for example, glucocorticoid-induced). Reports were excluded if the subjects had prior treatment with PTH or a PTH analogue or treatment with bisphosphonates within the previous 12 months. Both area BMD data measured by dual X-ray absorptiometry (DXA) and volumetric BMD data measured by quantitative controlled trials were eligible. The Jadad scale was used to assess the quality of included randomly controlled trials (RCTs), where a score of <3 indicated low quality [19].

### Statistical analysis

Changes in BMD values were expressed in percent change vs. baseline for both PTH and bisphosphonate treatment groups. We calculated the weighted mean differences (WMD) for percent changes in BMD. We conducted a random-effects model meta-analysis for heterogeneous outcomes and a fixed-effects model meta-analysis for homogeneous outcomes. The pooled analyses were performed using the Stata/SE 10.0 program for Windows (Stata Corporation, College Station, TX, USA). Statistical heterogeneity was investigated using the *χ* ^2^ test and *I* ^2^ statistic (*I* ^2^ represents the percentage of variability due to between-study variability). Funnel plots and the Egger's tests were used to estimate possible publication bias. A sensitivity analysis was conducted using the trim and fill method, to detect possible publication bias. A *p*-value less than 0.05 was considered to be statistically significant.

## Results

### Selected studies and characteristics

**Figure 1:**
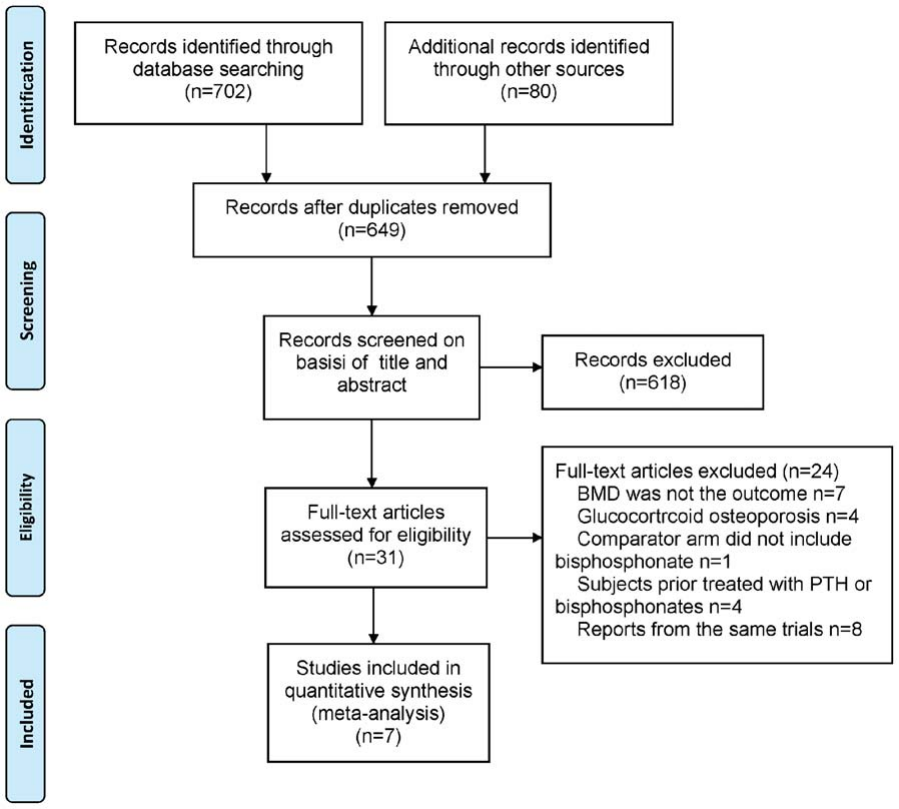
Flowchart of the meta-analysis.

A total of 782 potentially relevant citations were identified and screened, of which only 7 published RCTs met the inclusion criteria and were selected for this meta-analysis [10], [20], [21], [22], [23], [24], [25] (Figure 1). The main characteristics of the 7 studies are shown in Table 1. The level of evidence for each article was graded from scores 3 to 5 according to the Jadad quality score. A total of 944 patients, 896 women and 48 men were included in this analysis. Six trials involved postmenopausal women with osteoporosis [10], [20], [21], [22], [24], [25] and one trial involved osteoporotic men [23]. Of the 7 included trials, 5 had demonstrated the effects of alendronate [10], [20], [22], [23], [24], one showed results from risedronate [21] and the last showed the effects of zoledronic acid [25]. Allocation concealment was adequately reported in 4 trials [19], [20], [22], [24] and unclear in the remaining trials. Four trials were double-blind or partially double-blind [10], [22], [24], [25].

**Table 1:** Basal characteristics of clinical trials enrolled in the analysis.

Author (Ref.)	Year	Gender(F/M)	Number of patients (PTH/bisphosphonate)	Intervention (calcium and/or vitamin D)	Duration (months)	Outcomes measured: Areal BMD, volumetric BMD	Jadad score [19]
Cosman et al. [25]	2011	F	138 vs.137	20 µg PTH (1–34)/day + placebo infusion of zoledronic acid vs. a single intravenous infusion of zoledronic acid 5 mg. No placebo PTH. All: 1000–1200 mg calcium/day +400–800 IU vitamin D/day	12	Areal BMD: lumbar spine, total hip, femoral neck	3
Finkelstein et al. [20]	2010	F	20 vs. 29	40 µg PTH (1–34)/day vs. alendronate 10 mg/day. No placebo PTH. All: 1000–1200 mg calcium/day (diet or suppl.) +400 IU vitamin D/day	30	Areal BMD: lumbar spine, total hip, femoral neck, distal radius. Volumetric BMD: lumbar spine.	3
Anastasilakis et al. [21]	2008	F	22 vs. 22	35 mg Risedronate/week vs. 20 µg PTH (1–34)/day. No placebo PTH. All: 500 mg elemental calcium/day+400 IU vitamin D/day	12	Areal BMD: lumbar spine.	3
McClung et al. [22]	2005	F	102 vs. 101	20 µg PTH (1–34)/day+oral placebo vs. 10 mg alendronate 10/day+placebo injection. All: 1000 mg calcium/day (diet or suppl.) +400 to 800 IU vitamin D/day	12	Areal BMD: lumbar spine and femoral neck. Volumetric BMD: lumbar spine	3
Finkelstein et al. [23]	2003	M	20 vs. 28	40 µg PTH (1–34)/day vs. alendronate 10 mg/day. No placebo PTH. All: 1000 to 1200 mg calcium/day (diet or suppl.) +400 IU vitamin D/day	30	Areal BMD: lumbar spine (posteroanterior and lateral), total hip, femoral neck, distal radius. Volumetric BMD: lumbar spine.	3
Black et al. [10]	2003	F	119 vs. 60	100 µg PTH (1–84)/day vs. alendronate 10 mg/day. Placebo PTH All: 500 mg calcium/day+400 IU vitamin D/day	12	Areal BMD: lumbar spine, total hip, femoral neck, distal radius . Volumetric BMD: lumbar spine	4
Body et al. [24]	2002	F	73 vs. 73	40 µg rPTH (1–34) +oral placebo vs. alendronate+placebo inj. All: calcium 1000 mg/day+vitamin D 400 to 1200 IU/day	14	Areal BMD: lumbar spine, total hip, femoral neck, distal radius	4

### Effects of PTH versus bisphosphonates on spinal BMD

**Figure 2:**
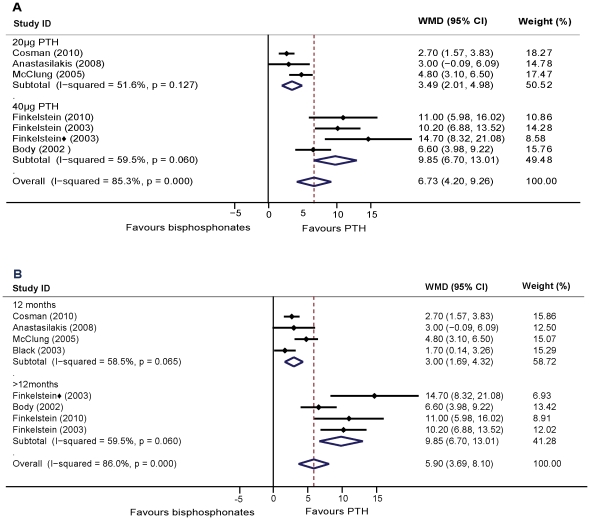
Assessment of the effects of PTH versus bisphosphonates on BMD of the lumbar spine. A: Subgrouped analysis of dosage of PTH (1–34) versus bisphosphonates treatment on spinal BMD. B: Subgrouped analysis of the duration of PTH (1–34) versus bisphosphonates treatment on spinal BMD. ⧫: The study reported BMD data in lateral spine.

**Figure 3:**
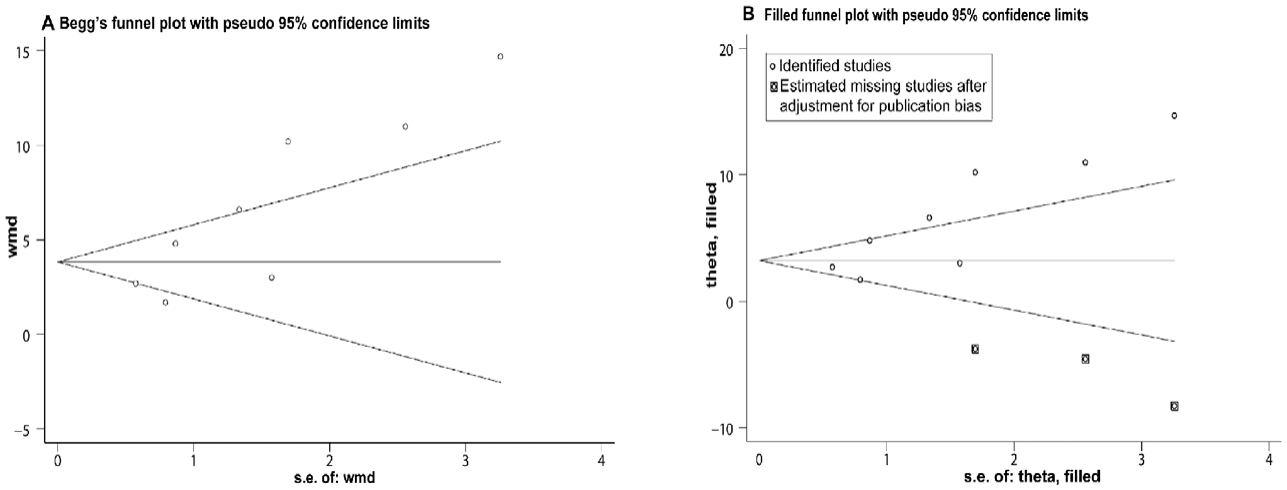
Funnel plot of all studies on the effects of PTH and bisphosphonates on spine BMD. A: Begg's funnel plot with pseudo 95% confidence limits. B: Filled funnel plot with pseudo 95% confidence limits. WMD, weighted mean difference; S.E., standard error.

All of the 7 RCTs studied lumbar spine areal BMD. Finkelstein et al. [23] reported BMD data in both posteroanterior spine and lateral spine. The pooled data showed that the percent change of increased BMD in the spine is higher with PTH in comparison to treatment with bisphosphonates after 12–30 months (WMD = 5.90, 95% CI: 3.69–8.10, *p*<0.01, n = 953). These estimates were heterogeneous. To explore this heterogeneity, we assessed the data within subgroups based on the gender of the participants and the type of agent used. For women, the pooled data from 6 studies showed that increases in BMD was higher in PTH treatment than that of bisphosphonates (WMD = 4.27, 95% CI: 2.46–6.08, *p*<0.01; n = 865). Whereas for men, Finkelstein et al. [23] reported that PTH treatment resulted in statistically significant increases in both posteroanterior spine and lateral spine BMD values as compared to that of alendronate. Comparing PTH treatment to alendronate, the increase in spine BMD was significantly higher in the PTH group (WMD = 7.42, 95% CI: 4.21–10.62, *p*<0.01; five studies, n = 649). Comparing PTH values to other types of bisphosphonates, the results were consistent (WMD = 2.74, 95% CI: 1.68–3.74, *p*<0.01; two studies, n = 304). The effects of 20–40 µg of PTH (1–34) compared to those of the bisphosphonate groups showed a significantly higher increase in BMD values (WMD = 4.41, 95% CI: 3.60–5.21, *p*<0.01; six studies, n = 774). Heterogeneity remained in the above analysis. To further explore this heterogeneity, subgroup analysis showed that the effects of both 20 µg and 40 µg of PTH (1–34) treatment increased lumbar spine BMD values significantly higher than bisphosphonates (WMD = 3.31, 95% CI: 2.42–4.21, *p*<0.01; three studies, n = 491; WMD = 8.92, 95% CI: 7.10–10.75, *p*<0.01; three studies, n = 283, respectively). Heterogeneity was not found in both subgroups (Figure 2A). In addition, we also grouped the studies on the basis of the duration of treatment. PTH treatment has yielded consistently and significantly higher increments as compared to the bisphosphonate groups (12 months: WMD = 3.00, 95% CI: 1.69–4.32, *p*<0.01; four studies, n = 670; over 12 months: WMD = 9.85, 95% CI: 6.70–13.01, *p*<0.01; three studies, n = 283) (Figure 2B). Heterogeneity was not found in either duration subgroup. The shape of the funnel plot showed slight asymmetry and the Egger's test indicated publication bias (*P*<0.05) (Figure 3A). We conducted a trim and fill method to further investigate the publication bias. The imputed studies produced a symmetrical funnel plot (Figure 3B) and the pooled analysis incorporating the hypothetical studies continued to show statistically significant higher increments in BMD values with PTH treatment over those using bisphosphonates (WMD = 3.48, 95% CI: 1.09–5.88, *p*<0.01).

Four of the 6 RCTs reported volumetric BMD data of the lumbar spine. The overall pooled results by random-effects analysis showed that the increase was higher with PTH treatment compared to bisphosphonates during 12–30 months of follow-up (95% CI: 13.81–52.12, *p*<0.01; four studies, n = 325). This comparison showed significant heterogeneity (Table 2). Funnel plot and Egger's test results did not reveal signs of publication bias (plot not shown).

**Table 2:** Effects of PTH versus bisphosphonates on volumetric BMD of the spine.

Author (Ref.)	Volumetric BMD of the spine				Weight (%)	Weighted mean difference (WMD) of BMD (95%CI)
	PTH		bisphosphonates			
	n	percent change(%)	n	percent change(%)		
Finkelstein 2003 [23]	20	48.0±27.9	28	3.0±7.8	24.19	45.00 [32.44, 57.56]
Black 2003 [10]	119	25.3±15.1	60	10.3±8.1	26.78	15.00 [11.60, 18.40]
McClung 2005 [22]	26	19.0±17.3	23	3.8±16.3	25.35	15.20 [5.79, 24.61]
Finkelstein 2010 [20]	20	61.0±31.0	29	1.0±7.0	23.68	60.00 [46.18, 73.82]
Pooled	185		140		100	32.96 [13.81, 52.12]
Heterogeneity	Tau^2^ = 353.63; Chi^2^ = 56.19, df = 3; *I^2^* = 95% *p*<0.01					

### Effects of PTH versus alendronate on BMD of the hip

**Figure 4:**
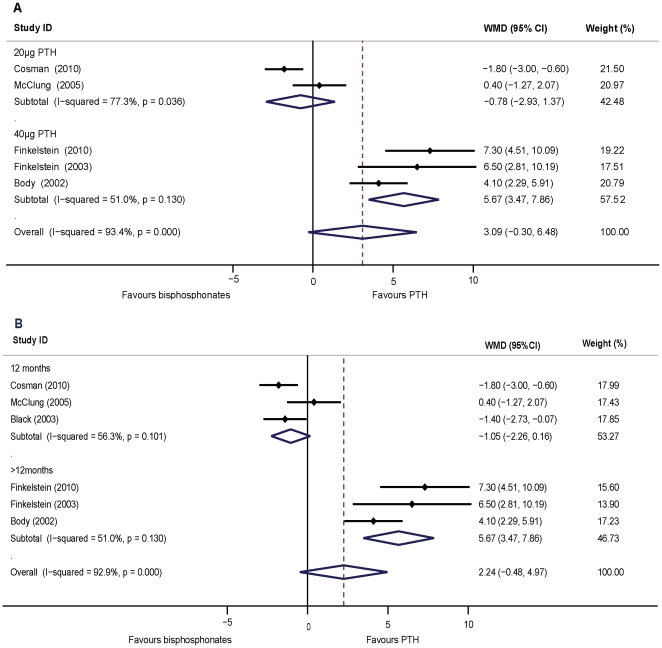
Assessment of the effects of PTH versus bisphosphonates on BMD of the femoral neck. A: Subgrouped analysis of the dosage of PTH (1–34) versus bisphosphonates treatment on the femoral neck BMD. B: Subgrouped analysis of the duration of PTH (1–34) versus bisphosphonates treatment on femoral neck BMD.

In this pooled analysis, the increases in the femoral neck BMD values were not significant between PTH and bisphosphonate treatments (WMD = 2.24, 95% CI: −0.48–4.97, *p* = 0.11, n = 824). In women, no significant difference was observed between the PTH and bisphosphonate groups (WMD = 1.54, 95% CI: −1.25–4.33, *p* = 0.28; five studies, n = 777). Nonetheless, statistical heterogeneity was large both in overall pooled analysis and in the analysis of women (*I* ^2^>80%, *p*<0.01). For men, there was only one trial (n = 47) that investigated the effects of 40 µg PTH (1–34) versus 10 mg alendronate daily on the femoral neck BMD during osteoporosis treatment [23]; therefore, we were unable to estimate a pooled effect. A sensitivity analysis excluding the trial using full-length PTH (1–84) [10] indicated that PTH (1–34) increased femoral neck BMD with no significant difference to the bisphosphonate group (WMD = 3.09, 95%CI: −0.30–6.48, *p* = 0.07, five studies, n = 645). Statistical heterogeneity was found in the analysis (*I* ^2^ = 93%, *p*<0.01). We then grouped the studies on the basis of the dose of PTH (1–34) used for treatment. 20 µg of PTH (1–34) yielded lower increments compared to bisphosphonates without statistical significance (WMD = −0.78, 95% CI: −2.93–1.37, *p* = 0.48; two studies, n = 403), while 40 µg PTH (1–34) showed significantly higher increments of BMD than alendronate (WMD = 5.67, 95% CI: 3.47–7.87, *p*<0.01 ; three studies, n = 242). Heterogeneity was found in the 20 µg treatment subgroup (*I^2^* = 77.3%, *p*<0.05), but not in the 40 µg subgroup (*I^2^* = 51.0%, *p* = 0.13) (Figure 4A). In the grouped studies comparing the duration of treatment, PTH treatment has yielded significantly higher increments than bisphosphonates with a duration of over 12 months (WMD = 5.67, 95% CI: 3.47–7.86, *p*<0.01; three studies, n = 242), and significantly lower increments at 12 months (WMD = −1.05, 95% CI: −2.26–0.16, *p*<0.01; three studies, n = 682). Heterogeneity was not found in either of the duration subgroups (*I* ^2^ = 51%, *p* = 0.13; *I* ^2^ = 56%, *p* = 0.09, respectively) (Figure 4B). The shape of the funnel plot showed slight asymmetry and the Egger's test indicated publication bias (*p*<0.05). The trimmed and filled funnel plot was symmetrical (plot not shown). The pooled analysis, incorporating the two hypothetical studies, continued to show statistically non-significant increments of BMD between the PTH and bisphosphonate groups (WMD = 0.01, 95% CI: −2.84–2.84, *p* = 0.10).

**Figure 5:**
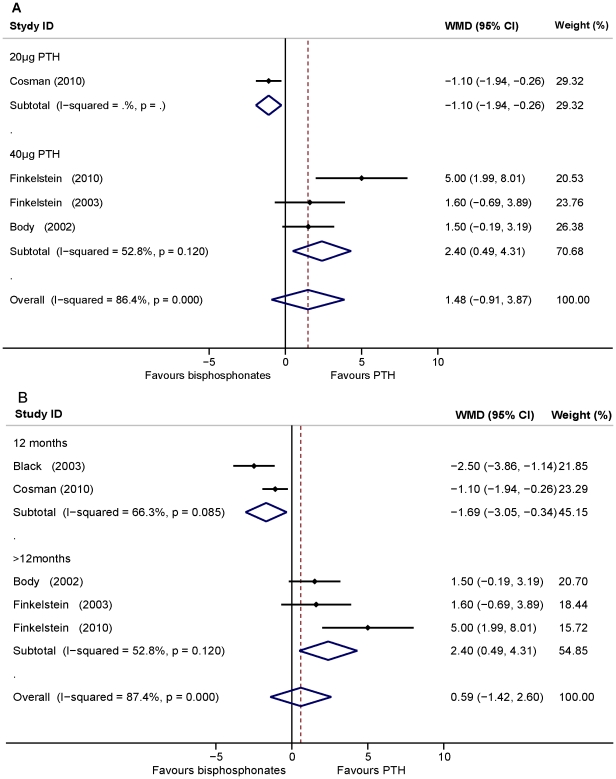
Assessment of the effects of PTH versus bisphosphonates on BMD of the total hip. A: Subgrouped analysis of the dosage of PTH (1–34) versus bisphosphonates treatment on total hip BMD. B: Subgrouped analysis of the duration of PTH (1–34) versus bisphosphonates treatment on total hip BMD.

The pooled data showed there was no significant difference between PTH and bisphosphonates in increasing total hip BMD (WMD = 0.59, 95% CI: −1.42–2.60, *p* = 0.57, five studies, n = 683). For PTH (1–34) treatment, the pooled data showed that BMD increase in the total hip was not significant as compared to bisphosphonates (WMD = 1.48, 95%CI: −0.91–3.87, *p*<0.05, four studies, n = 504). Heterogeneity was found among the studies. A sensitivity analysis conducted by removing the 20 µg PTH study [26] indicated that treatment with 40 µg of PTH (1–34) increased total hip BMD significantly higher than alendronate treatment (WMD = 2.40, 95%CI: 0.49–4.31, *p*<0.05, three studies, n = 242). Heterogeneity was not found among these studies (*I^2^* = 52.8%, *p* = 0.12) (Figure 5A). In addition, subgroup analysis indicated that PTH treatment for the duration of 12 months increased the total hip BMD significantly less than bisphosphonates while higher than bisphosphonates with a duration of over 12 months (WMD: −1.69, 95% CI: −3.05–0.34, *p*<0.05, two studies, n = 441; WMD = 2.40, 95% CI: 0.49–4.31, *P*<0.05, three studies, n = 242; respectively). Heterogeneity was not found in either subgroup (*I* ^2^ = 52.8%, *p* = 0.12; *I* ^2^ = 66.3%, *p* = 0.08, respectively) (Figure 5B). The shapes of the funnel plots showed symmetry and the Egger's test indicated the absence of publication bias (*p* = 0.14) (plot not shown).

### Effects of PTH versus alendronate on BMD of the distal radius

**Figure 6:**
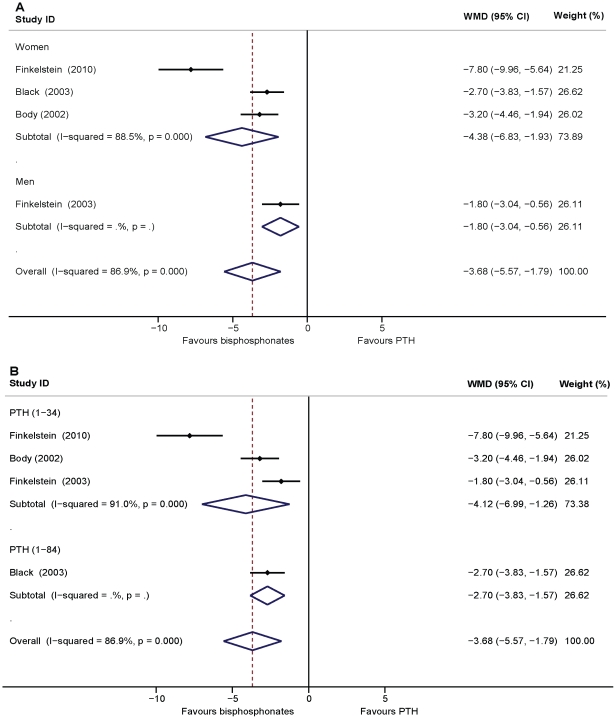
Assessment of the effects of PTH versus bisphosphonates on BMD of the distal radius. A: Subgrouped analysis of PTH versus bisphosphonates treatment on BMD of the distal radius in women and men. B: Subgrouped analysis of PTH (1–34) and PTH (1–84) versus bisphosphonates treatment on BMD of the distal radius.

A significant reduction in distal radius BMD was observed with PTH as compared to alendronate in the pooled analysis (WMD = −3.68, 95% CI: −5.57–1.79, *p*<0.01, four studies, n = 422). For the three studies involving women, the pooled effects presented the same result (WMD = −4.38, 95% CI: −6.83–1.93, *p*<0.01, n = 374). Heterogeneity was found in the above estimates. For PTH and alendronate treatment in men, data from the study by Finkelstein et al. [23] showed PTH decreased and alendronate increased BMD in the distal radius (n = 48, *p*<0.01) (Figure 6A). For PTH (1–34) treatment, the pooled data showed that PTH (1–34) significantly reduced BMD in the distal radius as compared to alendronate (WMD = −4.12, 95% CI: −6.69–1.26, p = 0.46, three studies, n = 243). The study by Black et al. [10] showed that distal radius BMD decreased with PTH (1–84) treatment, while BMD values increased with alendronate treatment (n = 179, *p*<0.01) (Figure 6B). The funnel plot did not reveal any signs of symmetry and the Egger's test indicated the absence of publication bias (*p* = 0.07) (plot not shown).

## Discussion

This meta-analysis revealed that therapy with PTH significantly increased both area and volumetric lumbar spine BMD as compared to treatment with bisphosphonates and PTH treatment induced duration- and dose-dependent increases of hip BMD as compared to bisphosphonates treatment. This analysis has also disclosed that for the distal radius, BMD was significantly lower from PTH treatment than alendronate treatment.

The overall results indicated that PTH therapy displayed higher gain in areal BMD than therapy with bisphosphonates in respect to dose and duration of PTH and gender of patients as supported by the BMD results by volumetric measurements. Previous trials concerning glucocorticoid-induced osteoporosis displayed similar results to our analyses. BMD of the lumbar spine had increased more than 2-fold with PTH (1–34) treatment compared to the alendronate treatment [27]. A former meta-analysis of pooled anti-resorptive comparative trials including alendronate showed significant reductions in PTH treated patients (1–34) for back pain, which was possibly caused by vertebral fracture [28]. Our analyses indicated that compared to low does of PTH treatment, greater BMD gains were obtained with high doses of PTH compared to treatment with bisphosphonates for the spine (WMD = 8.92 versus WMD = 3.31). This finding is in agreement with previous clinical trials involving PTH (1–34) [9], [29] and PTH (1–84) [12]. A former meta-analysis also disclosed a dose response relationship for BMD in the spine with both PTH (1–34) and PTH (1–84) treatments [30]. Previous placebo-controlled trials showed that spine BMD was significantly increased after 3 months of PTH therapy [12], [29]. Body et al. [24] revealed that the difference in lumbar spine BMD values between PTH (1–34) and alendronate treatment was statistically significant at 3 months. The alendronate-treated women required 12 months of treatment to increase lumbar spine BMD to a level equilvilant to women treated with PTH (1–34) for only 3 months.

In contrast to the spine, PTH treatment-induced changes in BMD of the hip were relatively inconsistent as compared to treatment with bisphosphonates. The former meta-analysis failed to draw a conclusion on the effect of PTH compared to bisphosphonates on hip BMD because of the low number of studies available [30]. In our meta-analysis, the overall analysis did not show significant differences between PTH and bisphosphonates. Former studies showed a trend towards higher hip BMD with higher PTH doses in both women and men [9], [12], [29]. Similarly, our subgroup analysis based on dosage of PTH (1–34) showed that compared to bisphosphonates, BMD increases of the femoral neck and total hip were significantly higher with treatment of high doses (40 µg/day) of PTH (1–34). On the other hand, analysis on the basis of duration indicated that the effect of PTH on hip BMD was inferior to bisphosphonates after treatment for 12 months, and was superior for over 12 months. This duration-related BMD change of PTH versus bisphosphonates could be explained by the different effects of PTH on trabecular and cortical bone. In trabecular bone, PTH adds new bone by increasing active bone remodeling units, which promotes new bone formation on quiescent bone surfaces. In cortical bone, PTH stimulates new bone formation mainly on the endocortical surface and to a lesser extent on the periosteal surface. One of the potential limitations is that it also increases intracortical (Haversian) remodeling and cortical porosity, thereby decreasing cortical BMD. Because the hip contains roughly equal amounts of cortical and trabecular bone, and BMD measured by DXA is a composite of these two bone types, the effect of PTH treatment on the hip BMD is the overall result of increased trabecular BMD and decreased cortical BMD. A transiently increased cortical remodeling space may lead to early reduction of cortical BMD. However, other competing effects within the periosteal, endocortical, and Haversian systems gradually allow the anabolic effects of PTH to predominate over the observed effects of an enlarged intracortical remodeling space. In this scenario, increased hip BMD during the second year of therapy showed a net gain in BMD.

In our meta-analysis, the distal radius was the only site at which the pooled data displayed BMD had decreased and was significantly lower in the PTH group as compared to the alendronate group. Previous observations disclosed that PTH therapy reduces BMD of the distal radius [9], [29]. On the other hand, in postmenopausal women, a significant correlation was observed between changes in percentage from baseline in bone strength of the ultradistal radius site in the alendronate treated group [31]. Using animal models, the decrease in areal density measured by DXA is likely due to increased Haversian remodeling. As compared to the hip, the distal radius consists mainly of cortical bone. Increased remodeling transiently increases cortical porosity, which does not affect biomechanical strength [32], [33]. In Neer's large placebo-controlled study, PTH therapy showed no increase in wrist fractures, which was consistent with the observations of preserved biomechanical strength in animal models [9].

For the present meta-analysis, most of the data originated from PTH (1–34) therapy, whereas the amount of data concerning PTH (1–84) is limited. Former studies showed a convincing reduction of vertebral fractures in both PTH (1–84) and PTH (1–34) treatment, but a reduction of non-vertebral fractures was shown in cases of treatment with PTH (1–34) only [34], [35]. No clear conclusions can be made about the potential differences in effectiveness on BMD of PTH (1–34) and PTH (1–84) because of the lack of directly comparative studies. The 100 µg PTH (1–84) trial should correspond to 43 µg of PTH (1–34) calculated on molecular basis [34]. We conducted grouped analysis with trials using 40 µg of PTH (1–34) and 100 µg PTH (1–84) both on the spine and hip BMD, but the results displayed large heterogeneity among trials. Thus, more data are needed on PTH (1–84) to establish if any clinically significant differences exist between these two types of PTH agents currently available. In our meta-analysis, dose and duration of PTH (1–34) was a main source of the heterogeneity. It was reported that adverse effects were more frequent in the 40 µg group compared to the 20 µg group [29]. Currently, the dose of PTH (1–34) approved by the US Food and Drug Administration is 20 µg. Even though BMD increased more with 40 µg for PTH treatment, fracture rates were similar [9]. Thus, only the lower dosage is approved for clinical administration. On the other hand, PTH (1–34) has been associated with the development of osteosarcoma in experimental animal models [36]; therefore, the safety of long-term clinical administration of PTH has yet to be determined. The use of PTH leads to new bone formation, but the skeletal response wanes over time, thereby limiting its anabolic effect. Sequential treatment with PTH and bisphosphonate showed benefits in maintaining gains in BMD [13], [37], [38].

Certain limitations in the present meta-analysis need to be addressed. First, the analysis is only based on published data and no unpublished data were included. Further, heterogeneity of the patients' ages and ethnic origin should be expected because it is impossible to match the cohorts completely for the analyses. On the other hand, our meta-analysis did not reveal gender-specific effects between PTH and bisphosphonates on BMD due to the limited number of trials on men. These factors limit the ability to elucidate the age-, ethnic- and gender- specific effects of PTH and bisphosphonates on BMD in osteoporosis treatment. Finally, the presented analysis was not designed to assess incident fractures.

We conclude that the bone-formation agent PTH substantially increased BMD of the lumbar spine compared to bisphosphonates as indicated in the current clinical reports on osteoporosis treatment. High doses of PTH (1–34) over 12 months of treatment duration increased BMD in the hip more effectively than bisphosphonates. PTH treatment reduced BMD of the distal radius significantly more than alendronate treatment. Further research could compare the effects of approved doses of PTH to bisphosphonates on BMD at vital sites in multicentric trials containing both women and men to attain robust clinical evidence.
